# High-fidelity simulation improves acute pain management competency in final-year medical students

**DOI:** 10.3389/fmed.2026.1907025

**Published:** 2026-09-04

**Authors:** Hassan Moria

**Affiliations:** Department of Surgery, University of Tabuk, Tabuk, Saudi Arabia

**Keywords:** acute pain management, clinical competence, high-fidelity simulation, medical education, multimodal analgesia, perioperative pain management, simulation-based learning, undergraduate medical education

## Abstract

**Background:**

Effective acute pain management requires integrating multimodal analgesia, clinical decision-making, and patient safety considerations. Although simulation-based education is increasingly used in medical training, its value for teaching complex acute pain management in undergraduate curricula has not been well established. This study evaluated whether high-fidelity simulation-based learning improved educational outcomes compared with traditional lecture-based teaching among final-year medical students.

**Methods:**

A quasi-experimental pre–post controlled study was conducted at the University of Tabuk Simulation Center in Saudi Arabia between September 2025 and February 2026. Final-year medical students were assigned, according to the academic teaching schedule, to either simulation-based learning or traditional lecture-based instruction. The simulation intervention included three clinically relevant acute pain scenarios. Outcomes included knowledge acquisition, assessed using a 15-item multiple-choice questionnaire; learner confidence, assessed using an 8-item Likert-scale questionnaire; and post-intervention clinical performance, assessed using a standardized competency-based checklist with scores ranging from 0 to 50. Between-group differences and pre–post changes were analyzed.

**Results:**

A total of 147 students were included in the final analysis: 73 in the simulation group and 74 in the traditional teaching group. Baseline demographic characteristics, knowledge scores, and confidence scores were comparable between groups (all *p* > 0.05). After the intervention, both groups improved; however, the simulation group showed greater gains in knowledge (12.8 ± 1.5 vs. 10.2 ± 1.8, *p* < 0.001), higher clinical performance scores (41.3 ± 3.8 vs. 34.0 ± 4.6, *p* < 0.001), and larger improvements in confidence (+9.8 vs. + 5.5 points, *p* < 0.001). Students reported high satisfaction with the simulation experience, with an overall satisfaction score of 4.7 ± 0.4. Effect size analysis showed a large effect for knowledge improvement (Cohen's *d* = 1.12) and a very large effect for clinical performance (Cohen's *d* = 1.58).

**Conclusions:**

Simulation-based training was associated with higher knowledge, confidence, and post-intervention performance scores. These findings should be interpreted in light of the quasi-experimental design, nonrandom allocation, and nonblinded assessment. The results support the use of simulation-based approaches in undergraduate pain education.

## Introduction

Pain is one of the most common reasons patients seek medical care and is a core concern across emergency departments, trauma units, surgical wards, and intensive care units. Acute pain, especially postoperative and trauma-related pain, remains highly prevalent among hospitalized patients and strongly influences patient satisfaction and clinical outcomes (1, 2). Poorly controlled acute pain has been linked to delayed recovery, impaired respiratory function, increased postoperative complications, longer hospitalization, and a higher risk of persistent chronic pain (3, 4). For these reasons, effective pain assessment and management are essential clinical competencies for physicians across specialties (5).

**Table 1:** High-Fidelity simulation scenarios used in the study.

Scenario	Clinical context	Key clinical features	Learning objectives	Expected analgesic strategy	Cognitive level (Bloom's taxonomy)
Scenario 1	Postoperative abdominal surgery with respiratory comorbidity	Severe postoperative pain following exploratory laparotomy in a patient with COPD	Assess severe postoperative pain, recognize respiratory risk, apply multimodal analgesia	Epidural analgesia when appropriate, multimodal systemic analgesia, cautious opioid use or IV PCA	Application/Analysis
Scenario 2	Trauma with rib fractures and pneumothorax	Severe chest pain due to rib fractures and flail chest	Recognize importance of analgesia for ventilation, apply regional techniques	Thoracic epidural or regional block (paravertebral/serratus) with multimodal analgesia	Application/Analysis
Scenario 3	Severe postoperative orthopedic pain after total knee replacement	Elderly patient with cardiovascular disease and prior CABG	Select safe analgesic strategies in medically complex patients	Peripheral nerve block (femoral/adductor canal), IV PCA when needed, avoid NSAIDs when contraindicated	Analysis/Evaluation

Regional anesthesia techniques were classified according to standard perioperative pain management principles.

**Table 2:** Outcome measures and data collection timeline.

Outcome	Instrument	Timepoint
Knowledge acquisition	15-item MCQ questionnaire	Pre- and post-intervention
Clinical performance	Structured scenario-based checklist	Post-intervention
Learner confidence	8-item Likert confidence scale	Pre- and post-intervention
Student satisfaction	Simulation evaluation survey	Post-simulation only

Instruments were developed by study investigators and validated through expert review and pilot testing.

**Table 3:** Structured clinical performance assessment checklist.

Domain	Assessed competency	Score range
Pain assessment	Ability to assess pain severity using validated scales and clinical examination	0–10
Clinical reasoning	Identification of risk factors and selection of appropriate analgesic strategy	0–10
Analgesic modality selection	Appropriate selection of epidural, nerve block, IV PCA, or PRN opioids	0–10
Patient safety	Recognition of contraindications and monitoring requirements	0–10
Communication & decision-making	Explanation of management plan and team/patient communication	0–10
Total Score		0–50

Assessment was performed using a standardized rubric by a trained instructor using identical clinical scenarios for both groups.

Despite advances in analgesic therapy and the availability of international guidelines for perioperative pain management, inadequate pain control remains a persistent global problem. Acute pain is often undertreated because of incomplete pain assessment, limited knowledge of analgesic pharmacology, and concerns about opioid-related adverse effects (6, 7). These challenges are especially important in patients with complex comorbidities, such as chronic respiratory disease, cardiovascular disease, renal dysfunction, or traumatic injuries, where clinicians must balance analgesic benefit against patient safety (8).

Modern perioperative care emphasizes multimodal analgesia, which combines pharmacologic and regional techniques with different mechanisms of action to improve pain control while limiting adverse effects (2, 9). This approach is central to Enhanced Recovery After Surgery (ERAS) protocols and current perioperative pain management strategies (10). Systemic analgesics, including acetaminophen, nonsteroidal anti-inflammatory drugs, and opioids, are commonly combined with regional anesthesia techniques (9, 11). Epidural analgesia is widely used for severe postoperative pain and may improve pulmonary outcomes (12), while peripheral nerve blocks, such as femoral nerve block and adductor canal block, are frequently used in orthopedic surgery to reduce opioid requirements and improve analgesia (13).

Opioid delivery strategies are also important in acute pain management. Intravenous patient-controlled analgesia (IV PCA) allows individualized opioid titration within safety limits and is associated with improved patient satisfaction (14). In contrast, intermittent “as-needed” (PRN) opioid administration may delay analgesic delivery and lead to inconsistent pain control (15). Understanding these approaches is essential for safe and effective clinical practice.

Despite the clinical importance of these skills, undergraduate pain education remains uneven across medical schools. Studies have demonstrated variation in both the quantity and quality of pain-related teaching, with medical students often reporting gaps in analgesic pharmacology, multimodal pain strategies, safe opioid prescribing, and confidence in managing acute pain in clinical settings (16–19). These gaps are concerning because newly graduated physicians are often expected to make early decisions about acute pain management in a range of clinical settings.

Traditional lecture-based teaching remains common in undergraduate medical education. Lectures can efficiently transmit core knowledge; however, they may be less effective at developing clinical reasoning, situational awareness, and decision-making in complex scenarios (21). Acute pain management requires students to integrate pain severity, comorbidities, pharmacologic considerations, and treatment-related risks, creating a need for educational methods that allow learners to apply knowledge in clinically realistic contexts.

Simulation-based learning offers one way to connect theoretical knowledge with clinical practice. It allows learners to manage realistic clinical scenarios, make decisions in a safe environment, and develop clinical reasoning skills without risking patient safety (20, 22). High-fidelity simulation uses advanced mannequins capable of reproducing physiological responses and clinical deterioration, thereby increasing realism and learner engagement (20, 23, 24). Educational theory also supports simulation-based learning through experiential models that emphasize active participation, reflection, and feedback (24, 25).

Evidence from several medical disciplines suggests that simulation-based education can improve knowledge, clinical competence, and learner confidence (22, 26, 27). Structured debriefing also supports reflective learning and clinical reasoning (26, 27). In pain education, however, the evidence remains limited. Many studies focus on postgraduate trainees or assess isolated outcomes, such as knowledge or satisfaction, rather than combined measures of knowledge, confidence, and clinical performance in undergraduate learners (27, 28).

A specific gap remains in the evaluation of simulation-based learning for undergraduate acute pain management, particularly regarding multidimensional outcomes within structured curricula. In particular, limited evidence compares simulation-based learning with lecture-based teaching among final-year medical students, while assessing knowledge, learner confidence, and standardized performance-based outcomes (27, 28).

The present study addressed this gap by evaluating the association between high-fidelity simulation-based training and educational outcomes in acute pain management among final-year medical students during a surgical module at a single institution. The intervention used clinically relevant scenarios involving postoperative pain, trauma-related pain, and patients with significant comorbidities. Outcomes included pre–post knowledge, pre–post confidence, and post-intervention clinical performance measured with a standardized simulated assessment. The study provides context-specific evidence from a single institutional setting and supports further evaluation in larger, potentially randomized, educational designs.

The findings should be interpreted within the context of a single-institution implementation involving final-year medical students in a surgical module, which may limit generalizability to other settings or learner groups.

Acute pain management was selected because it requires students to integrate pharmacologic knowledge, principles of multimodal analgesia, and patient safety considerations in time-sensitive clinical situations. The simulation scenarios were designed to reflect real-world complexity and required students to select appropriate analgesic strategies, including epidural analgesia, peripheral nerve blocks, IV PCA, and PRN opioid administration.

Accordingly, the aim of this study was to evaluate whether high-fidelity simulation-based learning was associated with differences in knowledge, clinical performance, and learner confidence compared with traditional lecture-based teaching among final-year medical students.

We hypothesized that students exposed to simulation-based training would show greater improvements in knowledge and confidence, as well as higher post-intervention clinical performance scores, than students receiving lecture-based instruction.

## Methods

### Study design

This study used a quasi-experimental pre–post controlled design to evaluate high-fidelity simulation-based learning compared with traditional lecture-based instruction for teaching acute pain management to final-year undergraduate medical students.

The study is reported in accordance with the Transparent Reporting of Evaluations with Nonrandomized Designs (TREND) guidelines for nonrandomized evaluations of behavioral and educational interventions, adapted to quasi-experimental research in simulation-based medical education. The intervention was developed using principles of simulation-based medical education, including experiential learning, deliberate practice, structured facilitator-led debriefing, and competency-based assessment.

### Outcomes

The study evaluated primary and secondary educational outcomes to assess the intervention's effect. The primary outcomes were knowledge acquisition and clinical performance. The secondary outcomes were learner confidence and student satisfaction.

Knowledge acquisition was assessed with a validated 15-item multiple-choice questionnaire administered before and after the intervention. Learner confidence was evaluated with a structured 8-item confidence questionnaire before and after the intervention. Clinical performance was assessed after the intervention with a standardized competency-based performance checklist during high-fidelity simulation scenarios. Student satisfaction was assessed only in the simulation-based learning group after the intervention, using a structured questionnaire designed to evaluate the simulation learning experience.

### Study setting

The study was conducted at the Simulation Center of the University of Tabuk, Saudi Arabia, a medical education facility equipped with the SimMan® 4G high-fidelity patient simulator (Laerdal Medical, Stavanger, Norway). The center is designed to reproduce perioperative and trauma clinical environments. The simulator can generate dynamic airway, respiratory, cardiovascular, and neurological responses and provide real-time patient monitoring. These features allowed learners to assess changing clinical conditions, interpret physiological responses, and apply evidence-based pain management strategies in realistic scenarios.

The simulation intervention was designed in accordance with accepted principles of simulation-based medical education. In addition to high-fidelity physiological simulation, the program used standardized scenario delivery, clear learning objectives, structured facilitator-led debriefing, and competency-based performance assessment. These components support experiential learning, deliberate practice, reflective learning, and progressive development of clinical competence.

All simulation sessions were conducted in standardized, controlled environments to maintain consistency of educational delivery, simulation fidelity, and scenario reproducibility. The study took place between September 2025 and February 2026 during the surgical module of the final-year undergraduate medical curriculum.

### Participants

#### Study population

Final-year medical students enrolled in the surgical module at the University of Tabuk during the 2025–2026 academic year were eligible. A total of 152 students initially participated, including 84 females and 68 males. Those who declined participation, were absent during the intervention, had incomplete pre- or post-assessment data, or were missing outcome data were excluded. After exclusions, 147 students were included in the final analysis. All included participants provided written informed consent.

Baseline characteristics included age, sex, previous exposure to simulation-based education, and previous clinical rotations in anesthesiology or perioperative care. These variables were collected to assess comparability between the two educational groups and reduce the likelihood that differences in outcomes reflected preexisting group differences.

#### Sampling and sample size

All eligible students in the defined academic cohort were invited to participate. Because the intervention was embedded within the mandatory undergraduate curriculum, a census-based sampling approach was used to maximize cohort inclusion and reduce selection bias. No *a priori* sample size calculation was performed. Nevertheless, the final sample size provided sufficient statistical precision to detect moderate-to-large educational effects, consistent with previous simulation-based medical education studies.

#### Group allocation

Participants were allocated to either the simulation-based learning group or the traditional lecture-based teaching group according to the predefined academic timetable.

Randomization was not feasible because students were already assigned to fixed teaching groups within the institutional curriculum and scheduling structure. Thus, allocation was determined independently of investigator or student preference. Baseline comparability between groups was confirmed.

To maintain methodological consistency, instructional time, learning objectives, intended educational outcomes, and assessment methods were standardized across both groups. Thus, differences in outcomes were intended to reflect the mode of educational delivery rather than differences in content, teaching duration, or assessment procedures.

**Figure 1:**
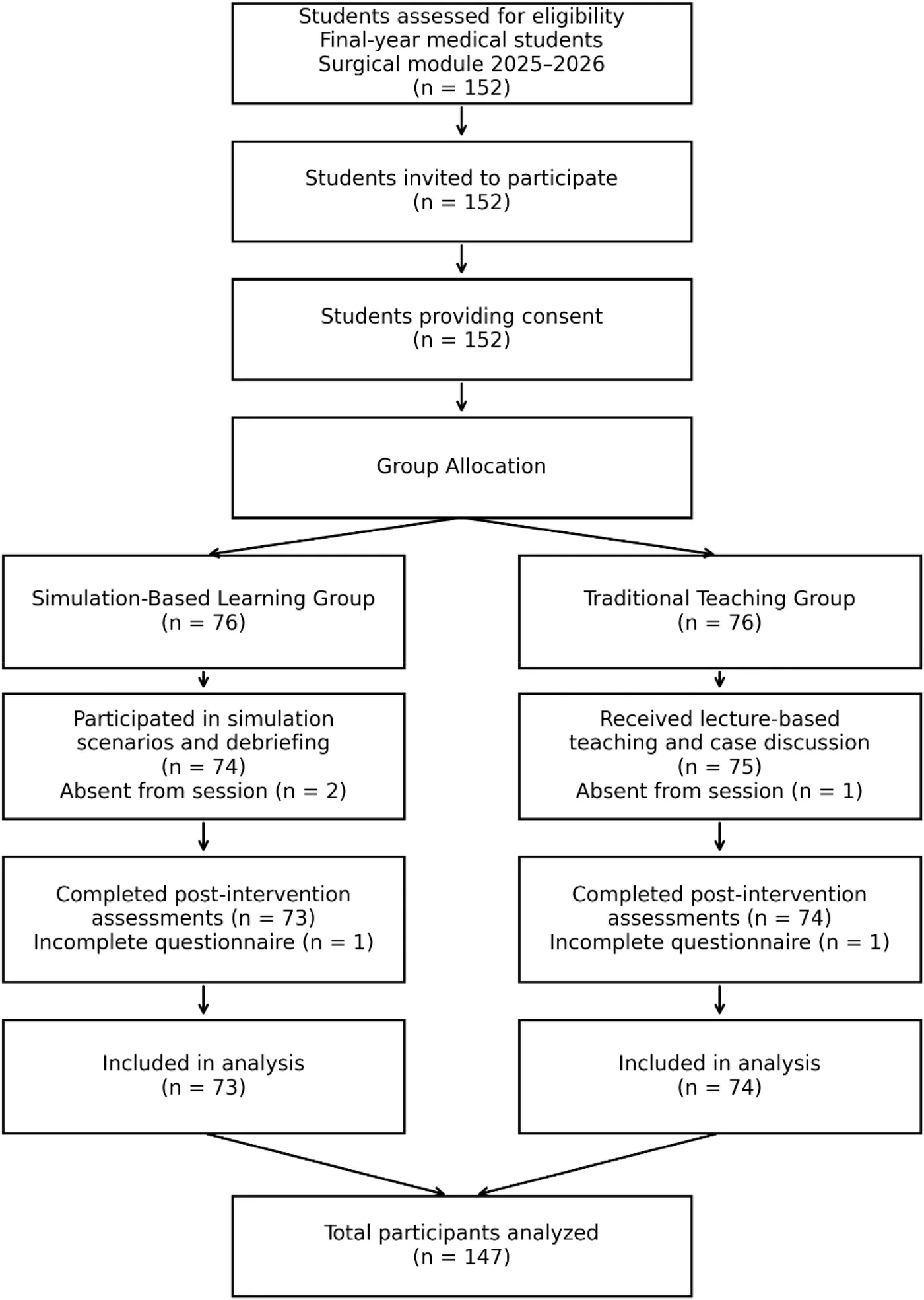
Flow diagram of participant recruitment, allocation to study groups, follow-up, and final analysis in the quasi-experimental study of simulation-based vs. traditional teaching in acute pain management.

A study flow diagram is presented in Figure 1.

#### Blinding and bias control

Blinding of participants and instructors was not feasible because of the nature of the educational intervention. Several steps were taken to reduce potential bias, including standardized teaching materials and scenario delivery, identical post-intervention assessments for both groups, standardized competency-based scoring rubrics, blinded statistical analysis of anonymized datasets, and equivalent instructional exposure time for all participants.

### Educational intervention

#### Core instruction

All participants received standardized theoretical instruction covering core principles of acute pain management, including pain assessment, multimodal analgesia, opioid pharmacology and safety, regional anesthesia techniques, intravenous patient-controlled analgesia (IV PCA), and pro re nata (PRN) opioid administration.

#### Simulation-Based learning group

Students in the simulation-based learning group completed three standardized high-fidelity simulation scenarios representing common but clinically challenging acute pain presentations in perioperative and trauma practice. The scenarios were developed according to principles of simulation-based medical education and aligned with predefined competency-based learning objectives.

Each simulation session consisted of a 5-min structured pre-briefing, a 15-min high-fidelity simulation scenario, and a 15-min facilitator-led structured debriefing.

Students participated in groups of five to six learners, allowing active participation, collaborative decision-making, and guided reflection. A single certified simulation instructor delivered all sessions to maintain consistency and minimize inter-instructor variability. The instructional design emphasized experiential learning, deliberate practice, structured reflection, and competency-based assessment.

#### Intervention fidelity

Intervention fidelity was maintained through standardized educational procedures throughout the study. These procedures included predefined scenario scripts, clearly specified competency-based learning objectives, standardized facilitator-led debriefing, delivery by a single certified simulation instructor, and pre-session faculty calibration to support consistent scenario implementation and learner assessment. No protocol deviations occurred during the study.

#### Simulation scenarios

Three standardized high-fidelity simulation scenarios were developed to represent common but clinically challenging acute pain presentations in perioperative and trauma practice: (1) postoperative abdominal surgery complicated by chronic obstructive pulmonary disease (COPD), (2) multiple rib fractures with pneumothorax, and (3) postoperative total knee replacement in a patient with significant cardiovascular disease.

Each scenario required learners to integrate pain assessment, individualized analgesic strategies, regional anesthesia techniques, opioid stewardship, and patient safety considerations while making evidence-based clinical decisions in realistic clinical environments. Core analgesic interventions included epidural analgesia, peripheral nerve blocks, IV PCA, and PRN opioid administration.

Detailed descriptions of the simulation scenarios, learning objectives, and expected learner actions are provided in Table 1.

The intervention included realistic high-fidelity scenarios, standardized scenario delivery, defined competency-based learning objectives, structured facilitator-led debriefing, and objective performance assessment. These elements are considered important features of simulation-based education because they support experiential learning, deliberate practice, reflection, and progressive clinical competence, rather than relying solely on simulation technology (24, 29, 36, 37).

#### Debriefing

Debriefing was conducted immediately after each simulation session using a standardized facilitator-led reflective model. The debriefing focused on clinical decision-making, evidence-based analgesic management, the application of multimodal pain management principles, and patient safety and risk recognition

Facilitated reflection encouraged learners to evaluate their clinical decisions, identify areas for improvement, and consolidate key learning objectives before progressing to later scenarios.

#### Control group

Participants in the control group received traditional lecture-based instruction supplemented by case-based discussions that covered the same predefined learning objectives and educational content as those in the simulation group. They did not participate in high-fidelity simulation scenarios or structured simulation debriefing.

### Outcome measures

#### Assessment timing

All educational outcomes were assessed immediately before and immediately after the educational intervention. No delayed follow-up assessment was conducted; thus, long-term knowledge retention and transfer of competence to later clinical practice were not evaluated. The outcome measures, corresponding assessment instruments, and data collection timepoints are summarized in Table 2.

### Assessment instruments

All instruments were specifically developed for this study and are provided in full in the Supplementary Files (File 1: Knowledge MCQs; File 2: Learner Confidence Scale; and File 3: Satisfaction Scale). Summaries are provided below.

### Knowledge assessment

Knowledge acquisition was assessed using a validated 15-item multiple-choice questionnaire that evaluated multimodal analgesia, opioid safety, regional anesthesia, and evidence-based clinical decision-making. Total scores ranged from 0 to 15, with higher scores indicating greater knowledge. Content validity was established through an independent expert panel review, and pilot testing confirmed the questionnaire's clarity and appropriateness. Internal consistency was acceptable (Cronbach's *α* = 0.81).

### Clinical performance

Knowledge acquisition was assessed using a validated 15-item multiple-choice questionnaire that evaluated learners' understanding of multimodal analgesia, opioid safety, regional anesthesia, and evidence-based clinical decision-making. Total scores ranged from 0 to 15, with higher scores indicating greater knowledge acquisition. Content validity was established through independent expert panel review, and pilot testing confirmed the clarity and appropriateness of the questionnaire. Internal consistency demonstrated acceptable reliability (Cronbach's *α* = 0.81). The assessed competencies, domains, and scoring structure are presented in Table 3.

### Learner confidence

Learner confidence was measured with a structured 8-item Likert-scale questionnaire. Total scores ranged from 8 to 40, with higher scores indicating greater confidence in acute pain management. The instrument showed good internal consistency (Cronbach's *α* = 0.84).

### Student satisfaction

Student satisfaction with the simulation-based educational experience was measured with a structured 6-item Likert-scale questionnaire. Total scores ranged from 6 to 30, with higher scores indicating greater satisfaction. The instrument showed good internal consistency (Cronbach's *α* = 0.86).

### Validity and reliability

All assessment instruments underwent a structured development and validation process before implementation. Content validity was established through independent expert panel review, yielding a Content Validity Index (CVI) greater than 0.90. Pilot testing with 12 students, who were excluded from the main study, evaluated clarity, relevance, difficulty, and completion time and led to minor wording refinements. Internal consistency was acceptable for all instruments (Cronbach's *α* = 0.81–0.86). Although construct validity was not formally evaluated, the instruments were aligned with the intervention's predefined educational objectives and competency framework.

### Pilot testing

Pilot testing assessed clarity, difficulty, and timing. Minor wording adjustments were made.

### Missing data handling

Missing data were reviewed before analysis and appeared to be random, with no apparent association with study group allocation. No imputation procedures were performed.

### Data management and verification

All data were independently checked for accuracy, cleaned before statistical analysis, cross-checked against original assessment records, and anonymized before statistical processing to protect data integrity and participant confidentiality.

### Statistical analysis

Statistical analyses were performed using IBM SPSS Statistics for Windows, Version 29.0 (IBM Corp., Armonk, NY, USA). Continuous variables are presented as mean ± standard deviation (SD), and categorical variables are presented as frequencies and percentages.

Between-group comparisons were performed using independent-samples *t* tests, and within-group pre–post comparisons were analyzed using paired-samples *t* tests. Categorical variables were compared using the chi-square test. Educational effect sizes were calculated using Cohen's *d* to estimate the magnitude of between-group differences beyond statistical significance. Analyses followed a complete-case approach, with participants who had incomplete outcome data excluded from the relevant analyses.

Normality of continuous variables was assessed using the Shapiro–Wilk test. Because the data were approximately normally distributed and parametric methods are robust for samples of this size, parametric analyses were considered appropriate.

Because this was an exploratory educational study with multiple educational outcomes, unadjusted two-tailed *p* values are reported. Although Bonferroni correction was considered, interpretation emphasized effect sizes alongside significance to provide a broader view of educational impact. Effect sizes were interpreted according to Cohen's conventional thresholds: *d* = 0.2, small; *d* = 0.5, medium; and *d* ≥ 0.8, large. A two-tailed *p* value < 0.05 was considered to indicate significance.

### Ethical considerations

This study was approved by the Research Ethics Committee of the University of Tabuk (Approval No. 645-405-2025; May 21, 2025). Participation was voluntary and had no effect on academic assessment, course grades, or progression within the medical curriculum. Written informed consent was obtained from all participants before enrollment. Participant confidentiality was maintained by anonymizing all datasets before statistical analysis, and study data were securely stored in accordance with institutional research governance requirements.

## Results

### Participant flow and study population

A total of 152 final-year medical students were assessed for eligibility. Of these, 147 students met the inclusion criteria and were included in the final analysis: 73 in the simulation-based learning group and 74 in the traditional lecture-based teaching group. All eligible students enrolled in the surgical module during the 2025–2026 academic year were invited to participate and provided informed consent before allocation.

Because the intervention was embedded within the existing curriculum, participants were assigned to study groups based on pre-scheduled teaching sessions, consistent with a quasi-experimental, nonrandomized design. Randomization was not used because of institutional scheduling constraints.

Attrition was minimal. In the simulation group, two students were absent during the intervention, and one had incomplete post-intervention data. In the traditional group, one student was absent, and one had incomplete questionnaire data. These cases were excluded from the final analysis according to predefined criteria.

Participant flow from recruitment to final analysis is illustrated in Figure 1.

### Participant baseline characteristics

Baseline demographic, academic, and prior exposure characteristics are summarized in Table 4. The two groups were well matched at baseline, with no significant differences observed.

**Table 4:** Baseline characteristics of participants.

Variable	Simulation Group (*n* = 73)	Traditional Group (*n* = 74)	*p*-value
Age (years), mean ± SD	24.40 ± 0.66	24.39 ± 0.66	0.89
Female, *n* (%)	41 (56.2)	43 (58.1)	0.81
Male, *n* (%)	32 (43.8)	31 (41.9)	—
Prior simulation exposure, *n* (%)	7 (9.6)	6 (8.1)	0.75
Prior anesthesia rotation, *n* (%)	2 (2.7)	1 (1.4)	0.58
Baseline knowledge score (0–15)	8.1 ± 1.9	8.0 ± 2.0	0.74
Baseline confidence score (8–40)	21.6 ± 4.1	21.3 ± 4.3	0.69

The mean age was nearly identical between the simulation and traditional groups (24.40 ± 0.66 years vs. 24.39 ± 0.66 years, *p* = 0.89). Sex distribution was also comparable, with females comprising 56.2% of the simulation group and 58.1% of the traditional group (*p* = 0.81).

Previous exposure to simulation-based education was low and similar in both groups (9.6% vs. 8.1%, *p* = 0.75). Previous anesthesia rotation experience was also comparable (2.7% vs. 1.4%, *p* = 0.58).

Baseline knowledge (8.1 ± 1.9 vs. 8.0 ± 2.0, *p* = 0.74) and baseline confidence (21.6 ± 4.1 vs. 21.3 ± 4.3, *p* = 0.69) scores did not differ significantly between groups.

Overall, the absence of significant baseline differences supports group comparability before the intervention.

### Knowledge acquisition outcomes

**Figure 2:**
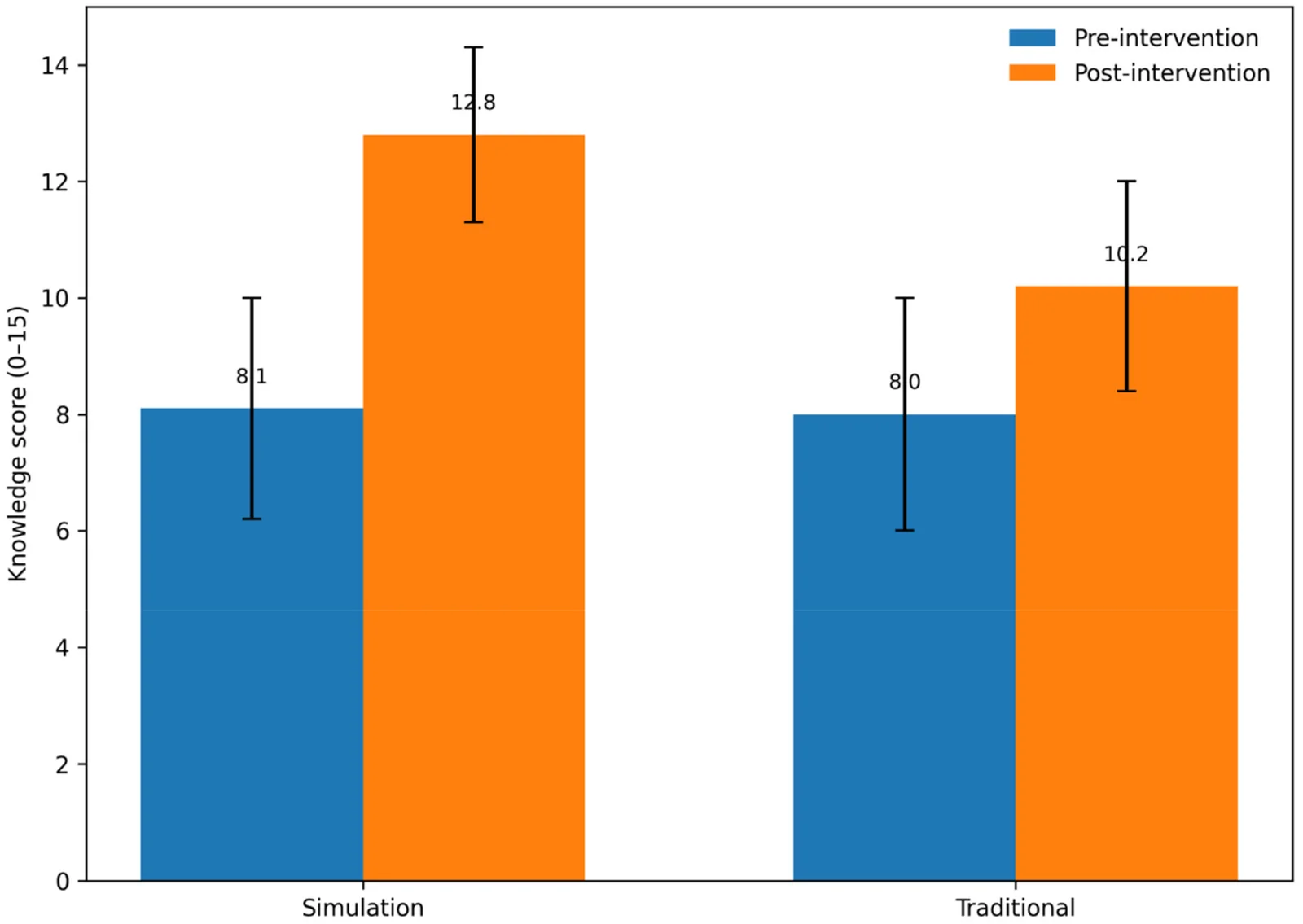
Comparison of knowledge scores before and after the educational intervention in simulation-based and traditional teaching groups. Bars represent mean values and standard deviations. Statistical analysis used paired and independent *t*-tests.

Knowledge outcomes are presented in Table 5 and Figure 2.

**Table 5:** Knowledge outcomes before and after intervention.

Outcome	Simulation	Traditional	*p*-value
Baseline knowledge	8.1 ± 1.6	8.0 ± 1.7	0.82
Post-intervention knowledge	12.8 ± 1.5	10.2 ± 1.8	<0.001
Knowledge improvement	+4.7 ± 1.8	+2.2 ± 1.6	<0.001

Mean differences were analyzed using independent and paired *t*-tests. No adjustment for multiple comparisons was applied (exploratory domain analysis).

At baseline, knowledge scores were comparable between groups (simulation: 8.1 ± 1.6 vs. traditional: 8.0 ± 1.7; *p* = 0.82). Following the intervention, both groups demonstrated improvement; however, gains were significantly greater in the simulation group. The post-intervention knowledge scores in the simulation and traditional groups were 12.8 ± 1.5 and 10.2 ± 1.8, respectively (*p* < 0.001); the mean improvement in the simulation group was +4.7 ± 1.8, and that in the traditional group was +2.2 ± 1.6, and this difference was significant (*p* < 0.001).

These findings indicate greater knowledge acquisition after high-fidelity simulation-based learning.

### Clinical performance outcomes

**Figure 3:**
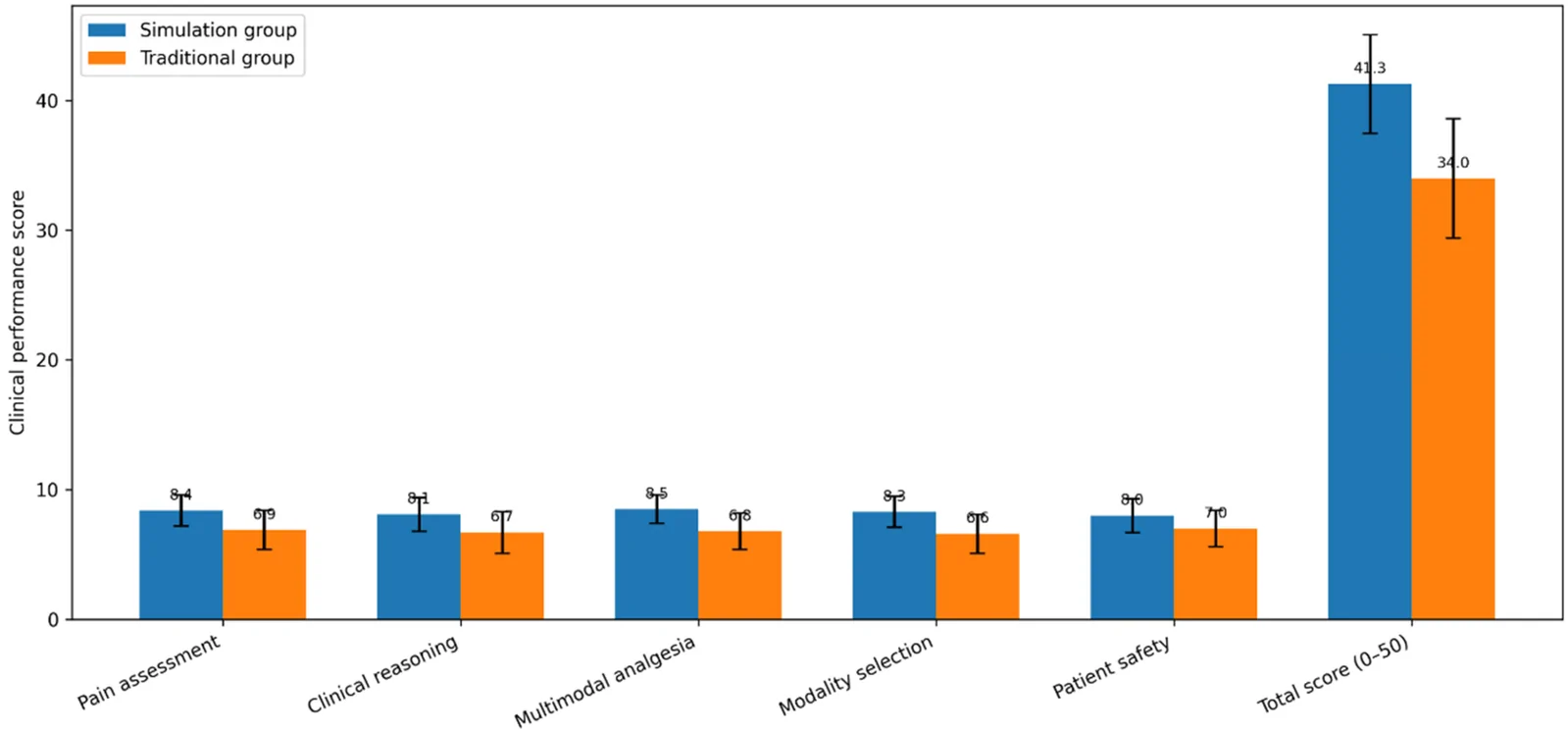
Comparison of clinical performance across assessment domains between simulation-based and traditional teaching groups. Bars represent mean scores with standard deviations. Simulation-trained students demonstrated superior performance across all domains.

Clinical performance outcomes are summarized in Table 6 and Figure 3. Students in the simulation-based learning group achieved significantly higher scores than those in the traditional teaching group across all assessed performance domains. Scores were higher for pain assessment (8.4 ± 1.2 vs. 6.9 ± 1.5, *p* < 0.001), clinical reasoning (8.1 ± 1.3 vs. 6.7 ± 1.6, *p* < 0.001), multimodal analgesia application (8.5 ± 1.1 vs. 6.8 ± 1.4, *p* < 0.001), analgesic modality selection (8.3 ± 1.2 vs. 6.6 ± 1.5, *p* < 0.001), and patient safety (8.0 ± 1.3 vs. 7.0 ± 1.4, *p* = 0.002).

**Table 6:** Clinical performance outcomes.

Domain	Simulation Group (*n* = 73)	Traditional Group (*n* = 74)	*p*-value
Pain assessment	8.4 ± 1.2	6.9 ± 1.5	<0.001
Clinical reasoning	8.1 ± 1.3	6.7 ± 1.6	<0.001
Multimodal analgesia application	8.5 ± 1.1	6.8 ± 1.4	<0.001
Analgesic modality selection	8.3 ± 1.2	6.6 ± 1.5	<0.001
Patient safety	8.0 ± 1.3	7.0 ± 1.4	0.002
Total score (0–50)	41.3 ± 3.8	34.0 ± 4.6	<0.001

The overall clinical performance score was significantly higher in the simulation group than in the traditional teaching group (41.3 ± 3.8 vs. 34.0 ± 4.6, *p* < 0.001). These findings suggest that simulation-based learning improved students' ability to assess pain, apply clinical reasoning, use multimodal analgesia strategies, select appropriate analgesic modalities, and incorporate patient safety principles into acute pain management.

### Learner confidence outcomes

Learner confidence findings are summarized in Table 7. Baseline confidence scores were comparable between the simulation-based learning and traditional teaching groups (21.6 ± 4.1 vs. 21.3 ± 4.3, *p* = 0.69). After the intervention, both groups showed significant improvements in confidence; however, the increase was greater among students who participated in simulation-based learning. Post-intervention confidence scores were significantly higher in the simulation group than in the traditional teaching group (31.4 ± 3.6 vs. 26.8 ± 4.0, *p* < 0.001), corresponding to mean improvements of +9.8 and +5.5 points, respectively (*p* < 0.001). These findings indicate that simulation-based learning was associated with greater improvement in students' confidence in managing acute pain and selecting appropriate multimodal analgesic strategies.

**Table 7:** Learner confidence outcomes.

Outcome	Simulation group (*n* = 73)	Traditional Group (*n* = 74)	*p*-value
Pre-confidence score	21.6 ± 4.1	21.3 ± 4.3	0.69
Post-confidence score	31.4 ± 3.6	26.8 ± 4.0	<0.001
Improvement score	+9.8 ± 3.2	+5.5 ± 3.0	<0.001

### Effect size analysis

Cohen's *d* was calculated for the principal educational outcomes to estimate the magnitude of between-group differences beyond statistical significance. Knowledge improvement showed a large effect size (*d* = 1.12), and the difference in clinical performance scores showed a substantial effect size (*d* = 1.58). These findings indicate that the benefits of high-fidelity simulation extended beyond statistical significance to include substantial educational gains in knowledge and clinical competence.

### Student satisfaction with simulation-based learning

Student satisfaction results are presented in Table 8. Satisfaction was assessed only among students in the simulation-based learning group (*n* = 73), because the questionnaire specifically evaluated the simulation experience and was not applicable to students receiving traditional lecture-based instruction.

**Table 8:** Student satisfaction with simulation-based learning (*n* = 73).

Item	Mean ± SD
Simulation improved understanding of acute pain management	4.6 ± 0.5
Improved clinical decision-making skills	4.5 ± 0.6
Simulation was realistic	4.7 ± 0.4
Improved multimodal analgesia understanding	4.6 ± 0.5
Debriefing enhanced learning	4.8 ± 0.4
Overall satisfaction	4.7 ± 0.4

**Scale:** 1 = strongly disagree, 5 = strongly agree.

Internal consistency of the instrument was good (Cronbach's *α* = 0.86).

Overall, students reported high satisfaction across all evaluated domains. The highest-rated aspect of the intervention was the structured debriefing session (4.8 ± 0.4), followed by overall satisfaction with the simulation experience (4.7 ± 0.4) and perceived realism of the simulation environment (4.7 ± 0.4). Students also reported that simulation improved their understanding of multimodal analgesia principles (4.6 ± 0.5), helped them connect theoretical knowledge with clinical practice (4.6 ± 0.5), and strengthened clinical decision-making skills (4.5 ± 0.6).

These findings indicate strong learner acceptance of the simulation-based intervention and indicate that students viewed high-fidelity simulation as a realistic, engaging, and educationally useful approach to learning acute pain management.

### Effect size and educational impact

The effect size analysis showed a large educational effect for knowledge improvement (Cohen's *d* = 1.12) and a very large effect for clinical performance (Cohen's *d* = 1.58). These findings suggest that the benefits of high-fidelity simulation are both significant and educationally meaningful.

## Discussion

### Principal findings

This quasi-experimental controlled study found that simulation-based learning was associated with greater improvement in acute pain management competency among final-year medical students than traditional lecture-based instruction. Students who completed the simulation intervention showed greater knowledge acquisition (12.8 ± 1.5 vs. 10.2 ± 1.8, *p* < 0.001), higher objective clinical performance scores (41.3 ± 3.8 vs. 34.0 ± 4.6, *p* < 0.001), and larger gains in confidence (+9.8 vs. + 5.5 points, *p* < 0.001). These differences were significant and educationally meaningful, as demonstrated by the large effect size for knowledge improvement (Cohen's *d* = 1.12) and the very large effect size for clinical performance (Cohen's *d* = 1.58). The magnitude of these effects suggests that the intervention may improve undergraduates' competence in complex tasks that require pain assessment, evidence-based pain management, clinical decision-making, and attention to patient safety.

The observed gains likely reflect the intervention's experiential, competency-based design rather than the use of simulation technology alone. Unlike lecture-based instruction, structured simulation required learners to integrate patient assessment, multimodal analgesic strategies, comorbidities, procedural options, and safety considerations while making time-sensitive decisions in realistic clinical environments. Realistic scenarios, deliberate practice, immediate feedback, and structured debriefing promote deeper cognitive processing, reflection, and transfer of knowledge into clinical practice, supporting the higher-order decision-making skills needed for effective pain management (24, 26, 27). Notably, the intervention aligned with best practices in simulation-based medical education, including three clinically distinct high-fidelity scenarios representing common but challenging perioperative and trauma-related pain presentations.

These scenarios included postoperative abdominal surgery complicated by chronic obstructive pulmonary disease, multiple rib fractures with pneumothorax, and severe postoperative pain after total knee replacement in a patient with significant cardiovascular comorbidity. Together, they required learners to integrate individualized analgesic strategies, patient-specific risk assessment, opioid stewardship, and patient safety principles across different clinical contexts. This provided a broader assessment of acute pain management competence than isolated procedural or pharmacologic tasks alone.

The present findings are consistent with growing evidence supporting simulation-based pain education. Recent studies of postoperative pain simulation, perioperative pain management education, and undergraduate simulation curricula have reported improvements in knowledge, clinical competence, confidence, and decision-making after structured simulation-based interventions (28, 30, 35). Whereas many of these studies focused on individual outcomes, the present study also demonstrated gains in objectively assessed clinical performance and reported large educational effect sizes, providing a more complete evaluation of undergraduate pain management competency.

Recent scoping and integrative reviews have similarly concluded that simulation-based education offers a useful framework for developing pain management competencies by integrating pharmacologic knowledge, procedural skills, patient safety principles, and structured reflection within clinically relevant learning environments (36, 37). Taken together, current evidence supports structured simulation-based education as an effective approach for developing competencies needed for evidence-based acute pain management.

An important contribution of this study is its multidimensional assessment of educational effectiveness. Previous pain education research has emphasized the importance of evaluating not only knowledge but also confidence and broader pain-management competencies (31, 32). Many previous simulation studies have focused mainly on knowledge, confidence, or satisfaction. In contrast, this study assessed cognitive, behavioral, and affective outcomes through standardized measures of knowledge, objective clinical performance, confidence, satisfaction, and effect size. The consistent improvements across these domains suggest that the intervention strengthened both theoretical understanding and the ability to apply evidence-based clinical reasoning in acute pain management.

Beyond the use of high-fidelity technology, the intervention incorporated clinically relevant scenarios, structured facilitator-led debriefing, deliberate practice, and competency-based performance assessment. These components are widely recognized as key features of strong simulation-based curricula (24, 29, 36, 37). The present study embeds these elements in acute pain scenarios, adding to the evidence supporting structured simulation-based education as a competency-based approach to improving undergraduate pain management training.

### Simulation-Based education and clinical competence

The substantial improvement in clinical performance after high-fidelity simulation-based learning suggests that its educational value extends beyond factual knowledge to applied clinical competence. Compared with lecture-based teaching, simulation required students to assess pain severity, interpret patient-specific information, select evidence-based analgesic strategies, anticipate complications, and justify decisions within realistic, time-sensitive scenarios. These processes closely resemble clinical practice and require integration of knowledge, decision-making, and patient safety principles.

From an educational perspective, these findings are consistent with experiential learning theory, which emphasizes active participation, reflective observation, and structured feedback as mechanisms for transforming knowledge into competence (6). High-fidelity simulation applies these principles by allowing learners to participate in immersive clinical experiences, followed by structured, facilitator-led debriefings. This process helps learners recognize errors, refine decisions, and consolidate learning before applying these concepts in subsequent scenarios. The high learner ratings for debriefing quality in this study further support the value of guided reflection in simulation-based education.

Current evidence supports this approach. Undergraduate simulation studies have demonstrated that structured simulation-based training improves knowledge, clinical decision-making, procedural competence, and self-efficacy compared with conventional educational methods, especially when clinically relevant scenarios are paired with standardized debriefing and competency-based assessment (22, 26, 27, 30, 35). Scoping and integrative reviews also conclude that simulation provides a safe and effective environment for developing pain assessment, procedural skills, clinical reasoning, and clinical competence through deliberate practice and structured feedback. A scoping review of regional anesthesia education similarly highlights the value of simulation for improving procedural competence and learner confidence across educational settings (24, 36, 37). Additional educational research has demonstrated that simulation-based methods can improve pain assessment skills and analytical reasoning through learner-centered scenarios that mirror clinical practice (38, 39).

Notably, the simulation intervention was aligned with current recommendations for simulation-based medical education. In addition to high-fidelity physiological simulation, the intervention used standardized scenario delivery, structured facilitator-led debriefing, predefined competency-based learning objectives, and objective performance assessment. These elements support deliberate practice, reflection, and the development of progressive competence (24, 29, 36, 37). Overall, the findings suggest that the effectiveness of simulation depends on both technology and the combination of relevant scenarios, active engagement, deliberate practice, structured reflection, and immediate feedback. This approach is well suited to acute pain management, where effective clinical performance requires simultaneous integration of pharmacologic knowledge, multimodal strategies, patient-specific risk assessment, and patient safety during complex perioperative and trauma-related decision-making.

### Multimodal pain management and patient safety

Acute pain management is cognitively demanding because clinicians must integrate pain assessment, comorbidities, multimodal analgesic options, procedure-specific factors, and potential adverse events when selecting treatment. Clinical guidelines consistently recommend multimodal analgesia as the standard of care, emphasizing combined pharmacologic and regional techniques to optimize pain control, reduce opioid use, and limit treatment-related complications (2, 5, 7, 9, 33). Thus, undergraduate medical education needs learning environments that go beyond didactic instruction to help students develop these decision-making skills.

The scenarios used in this study were designed to reflect clinical situations requiring students to integrate epidural analgesia, peripheral nerve blocks, IV PCA, and individualized opioid prescribing while considering patient-specific risks. Students in the simulation group performed better across all domains of clinical assessment, including pain assessment, clinical reasoning, application of multimodal analgesia, analgesic modality selection, and patient safety. These findings suggest that simulation helped students connect theoretical knowledge with clinical decision-making rather than simply recall pharmacologic facts.

Recent educational studies have also demonstrated that simulation-based pain management training can improve learners' ability to assess pain systematically, prioritize analgesic interventions, and apply evidence-based multimodal strategies in clinical contexts (22, 27, 30, 35). Educational approaches using simulated or virtual clinical environments have also been applied to opioid-related decision-making and morphine titration training among medical students (34). A recent randomized educational trial found that simulation-based instruction led to greater procedural competence than conventional teaching for ultrasound-guided regional anesthesia, further supporting the role of simulation in developing complex pain-related procedural and decision-making skills (23). Reviews of undergraduate pain simulation education also conclude that clinically authentic scenarios promote higher-order reasoning by requiring learners to synthesize pharmacologic knowledge, procedural skills, communication, and patient safety within time-sensitive situations (24, 36, 37).

Collectively, these findings support simulation as an effective educational strategy for developing the clinical reasoning required for multimodal pain management.

Patient safety is also central to acute pain education. In this study, students who received simulation-based training performed better in recognizing opioid-related respiratory risk, identifying contraindications to analgesic techniques, and selecting safer strategies for patients with significant comorbidities. These improvements are particularly relevant given the growing emphasis on opioid stewardship, individualized prescribing, and perioperative risk reduction within enhanced recovery pathways (2, 5, 9, 33). Simulation provides a safe setting in which learners can recognize, analyze, and correct potentially harmful decisions without exposing patients to risk, thereby strengthening clinical competence and safety awareness (22, 36).

Taken together, these findings suggest that high-fidelity simulation offers advantages beyond improving factual knowledge. Simulation recreates realistic clinical complexity in a controlled environment, thereby helping learners integrate multimodal analgesia, patient safety, and evidence-based reasoning into coherent decisions that more closely resemble real acute pain management.

### Comparison with previous simulation studies

The educational outcomes observed in this study are broadly comparable with those reported in recent undergraduate simulation-based pain education programs. Similar improvements in knowledge, confidence, and clinical decision-making have been reported after structured simulation-based training in postoperative pain management, perioperative care, and acute pain education (22, 27, 30, 35). More recently, a randomized educational trial comparing simulation-based and conventional teaching for ultrasound-guided regional anesthesia showed greater procedural competence among learners receiving simulation-based instruction, further supporting the use of simulation-based education for complex pain-related competencies (23). However, many previous studies primarily assessed single outcomes, such as knowledge, self-efficacy, satisfaction, or procedural performance. The present study demonstrated improvements across knowledge acquisition (12.8 ± 1.5 vs. 10.2 ± 1.8, *p* < 0.001), objective clinical performance (41.3 ± 3.8 vs. 34.0 ± 4.6, *p* < 0.001), and learner confidence (+9.8 vs. + 5.5 points, *p* < 0.001), with large effect sizes.

These findings are supported by scoping and integrative reviews showing that structured simulation enhances clinical competence through active participation, deliberate practice, repeated decision-making, and reflective debriefing in clinically relevant learning environments (24, 29, 36, 37). Similar gains have been reported in postoperative pain simulation programs, in which learners improved confidence, pain assessment, and evidence-based analgesic decision-making following structured simulation activities (30, 35). Overall, the current evidence supports structured simulation-based education as an effective strategy for strengthening competency in pain medicine and acute pain management.

Notably, this study employed acute pain scenarios that required learners to integrate multimodal pain management, regional anesthesia options, opioid stewardship, patient-specific risk assessment, and patient safety when making individualized treatment decisions. Rather than focusing on isolated procedural skills or a single educational outcome, we assessed multiple complementary domains using standardized performance assessment. This multidimensional approach provides a broader evaluation of undergraduate pain medicine training and supports the integration of structured simulation-based education into competency-based undergraduate curricula.

### Educational implications

The findings have important implications for undergraduate medical education and curriculum development. As health care systems increasingly adopt competency-based educational frameworks, traditional lecture-based teaching alone may not prepare medical students for the complexity of clinical decision-making. Acute pain management is a challenging area of practice because students must integrate pharmacologic knowledge, evidence-based analgesic strategies, patient-specific risk assessment, communication, and safety considerations within time-sensitive clinical environments. The present findings suggest that high-fidelity simulation can help students develop these competencies before they encounter similar situations in clinical practice (12, 22, 36).

The educational value of simulation extends beyond factual knowledge. Simulation promotes the cognitive, psychomotor, and behavioral competencies that support effective clinical performance by exposing learners to clinical scenarios requiring active decision-making. Structured debriefing reinforces reflective learning by helping students evaluate their decisions, recognize errors, and refine future practice. These processes are central to competency-based medical education and support progression from knowledge acquisition to supervised clinical practice (22, 24, 36).

The simulation scenarios in this study addressed common but complex situations encountered in surgical and perioperative care, including postoperative abdominal surgery complicated by respiratory comorbidity, traumatic rib fractures with pneumothorax, and severe postoperative orthopedic pain in a patient with cardiovascular disease. These scenarios required learners to apply multimodal pain management principles, select appropriate analgesic modalities, recognize opioid-related risks, and prioritize patient safety while making individualized treatment decisions. Such clinical experiences are difficult to reproduce through classroom teaching alone and may improve readiness for supervised clinical practice.

From a curricular perspective, these findings support the integration of structured high-fidelity simulation into undergraduate teaching modules in surgery, anesthesiology, emergency medicine, and perioperative care. This recommendation is consistent with recent evidence on the expanding role of simulation-based training in regional anesthesia education and broader anesthesiology curricula (24). Simulation should complement, not replace, didactic teaching by allowing students to apply theoretical knowledge in clinical contexts and receive immediate formative feedback through debriefing. The value of this approach is supported by high learner satisfaction in the present study, with an overall satisfaction score of 4.7 ± 0.4 out of 5, as well as substantial improvements in knowledge acquisition (Cohen's *d* = 1.12) and clinical performance (Cohen's *d* = 1.58). These findings indicate that the intervention was well accepted and was associated with meaningful gains across cognitive, behavioral, and affective domains. High satisfaction may also promote engagement, participation, and motivation in experiential learning, supporting progressive competency development (35, 36). Thus, incorporating simulation-based pain management training into undergraduate curricula may improve clinical preparedness and support safe, evidence-based pain management among future physicians.

### Study strengths

This study has several methodological strengths. First, the controlled quasi-experimental design allowed direct comparison of high-fidelity simulation-based learning with traditional lecture-based instruction within the same undergraduate curriculum, with comparable baseline characteristics between groups. Second, educational effectiveness was assessed using a multidimensional framework that included objective knowledge testing, standardized clinical performance assessment, learner confidence, and student satisfaction, rather than relying on a single measure. Third, the simulation intervention used standardized, high-fidelity scenarios, structured, facilitator-led debriefings, and predefined performance checklists, thereby supporting consistency in delivery and assessment. In addition, the assessment instruments showed acceptable content validity and internal consistency. Finally, reporting both statistical significance and effect size estimates provides a clearer interpretation of educational impact, showing that the observed improvements were significant and educationally meaningful.

### Limitations

First, the quasi-experimental design without random allocation introduces the possibility of selection bias and limits causal inference, although baseline demographic characteristics, knowledge scores, and confidence scores were comparable between groups. Allocation according to the existing academic teaching schedule was necessary to preserve routine undergraduate instruction and reflects the practical realities of educational research within medical curricula.

Second, the study was conducted at a single academic institution, which may limit generalizability to undergraduate medical programs with different curricula, educational resources, or learner populations. Nevertheless, the intervention was integrated into a routine undergraduate surgical module, using educational principles broadly applicable to competency-based curricula, suggesting that similar benefits may be achievable in comparable settings.

Third, educational outcomes were assessed immediately after the intervention. Long-term retention of knowledge, clinical competence, and confidence was not evaluated. Thus, the durability of these gains and their transfer to later clinical practice remain uncertain. Future studies with delayed follow-up assessments, such as at 6 months or longer, are needed.

Fourth, clinical performance was assessed using standardized high-fidelity simulation scenarios rather than direct observation in real patient care. Although simulation-based assessment is a safe and widely used method for evaluating clinical competence, performance in simulated settings may not fully reflect workplace behavior or transfer to routine clinical practice.

Fifth, assessor blinding was not feasible because the same faculty member facilitated the simulation sessions and evaluated learner performance. Although standardized competency-based checklists and predefined scoring criteria were used to reduce observer bias, some measurement bias cannot be excluded.

Finally, because both groups belonged to the same academic cohort, students may have exchanged information outside scheduled teaching sessions, potentially contaminating the groups. Although no evidence of substantial contamination was identified, informal student-to-student information exchange could not be objectively measured and cannot be entirely ruled out.

Despite these limitations, we employed standardized educational procedures, validated assessment instruments, comparable baseline groups, and objective, competency-based outcomes, yielding a robust evaluation of simulation-based learning in undergraduate acute pain management education.

### Future research

Future research should confirm and extend these findings through multicenter studies involving diverse undergraduate medical programs to improve external validity and generalizability. Although randomized controlled educational designs may not always be feasible within routine curricula, future studies should use random allocation where practical to strengthen the evidence base.

Longitudinal studies with delayed follow-up assessments, such as at 6 months or longer, are needed to evaluate long-term retention of knowledge, clinical competence, and confidence acquired through simulation-based education.

Future research should also examine whether simulation-acquired competencies transfer to real clinical practice using workplace-based assessments or objective measures of clinical performance, and whether improvements in educational outcomes ultimately translate into better patient care.

Finally, economic evaluations of the cost-effectiveness, scalability, and long-term sustainability of high-fidelity simulation in undergraduate curricula would provide useful evidence for curriculum development, educational planning, and resource allocation.

## Conclusions

High-fidelity simulation-based learning produced significant and educationally meaningful improvements in knowledge acquisition, clinical competence, and learner confidence compared with traditional lecture-based instruction among final-year undergraduate medical students. The large effect sizes for knowledge improvement and clinical performance suggest that simulation-based education provides substantial benefits beyond conventional didactic teaching.

These findings support integrating structured, high-fidelity simulation into undergraduate medical curricula to strengthen competency in acute pain management and multimodal analgesia decision-making. Clinically realistic simulation scenarios may better prepare learners to apply evidence-based pain management principles, clinical decision-making, and patient safety considerations in complex clinical settings.

Future multicenter studies with longitudinal follow-up are warranted to determine whether competencies gained through simulation-based education are retained over time and whether these gains translate into sustained improvements in clinical practice and patient outcomes.

## Data Availability

The original contributions presented in the study are included in the article/Supplementary Material, further inquiries can be directed to the corresponding authors.
