# Supplementary material for: High-fidelity simulation improves acute pain management competency in final-year medical students
Source: Front Med (Lausanne). 2026 Sep 4;13:1907025. doi: 10.3389/fmed.2026.1907025 (PMC13585591; doi:10.3389/fmed.2026.1907025)
Supplement: Supplementary file 1 [file Supplementaryfile1.docx]

**SUPPLEMENTARY FILE 1**

**Acute Pain Management Knowledge Assessment Questionnaire (MCQs)**

**Study Title**

High-Fidelity Simulation Improves Acute Pain Management Competency in Final-Year Medical Students

**Purpose of the Instrument**

This questionnaire was developed to assess clinical reasoning and decision-making in acute pain management among final-year undergraduate medical students. It consists of 15 single-best-answer MCQs based on three clinical simulation scenarios.

It evaluates:

- Pain assessment
- Multimodal analgesia
- Opioid safety and monitoring
- Regional anesthesia techniques
- Adjuvant analgesics
- Patient-specific analgesic decision-making

All items are scenario-based and designed to assess application and analysis levels of cognition.

**Instrument Structure**

- Total items: 15 MCQs
- Score range: 0–15
- One correct answer per item

**Knowledge Assessment Questionnaire**

**Clinical Scenario 1**

**Postoperative Abdominal Surgery in a Patient with COPD**

A **68-year-old man with moderate chronic obstructive pulmonary disease (COPD)** undergoes exploratory laparotomy for bowel obstruction. Six hours postoperatively, he reports **severe abdominal pain (NRS 8/10)** and demonstrates shallow breathing due to pain. Oxygen saturation is **92% on 2 L/min via nasal cannula**.

**Question 1**

Which analgesic strategy would most effectively improve pain control **while minimizing the risk of opioid-related respiratory compromise**?

A. Repeated intravenous morphine boluses titrated to pain relief
B. Thoracic epidural analgesia using a low-dose local anesthetic–opioid combination
C. Intramuscular opioid administration every 4 hours
D. Scheduled oral NSAIDs alone

**Correct answer:** B
**Bloom’s level:** Application

**Question 2**

Thirty minutes after initiating **IV PCA morphine**, the patient becomes progressively drowsy and his respiratory rate falls to **9 breaths/min**. Oxygen saturation is **90%**.

What is the **most appropriate immediate management step**?

A. Increase supplemental oxygen and continue the PCA unchanged
B. Temporarily stop PCA, support ventilation, and titrate naloxone as needed
C. Reduce the PCA bolus dose and continue close observation only
D. Administer a benzodiazepine to reduce distress

**Correct answer:** B
**Bloom’s level:** Analysis

**Question 3**

Which statement best explains the rationale for using **multimodal analgesia** in this postoperative patient?

A. It avoids the need for all opioid medications
B. It targets different mechanisms of pain, improving analgesia while reducing opioid requirements
C. It prevents all postoperative adverse events
D. It primarily improves pain by increasing sedation

**Correct answer:** B
**Bloom’s level:** Understanding

**Question 4**

Despite receiving **IV PCA morphine**, the patient continues to report **severe pain (NRS 8/10)**. His respiratory rate is **14/min**, and oxygen saturation is **94% on 2 L/min oxygen**.

Which of the following is the **most appropriate next step** to improve analgesia while minimizing opioid-related respiratory risk?

A. Increase the PCA morphine bolus dose and shorten the lockout interval
B. Add scheduled acetaminophen and assess suitability for thoracic epidural analgesia
C. Start a continuous opioid infusion in addition to PCA dosing
D. Administer intermittent benzodiazepines to reduce pain-related anxiety

**Correct answer:** B
**Bloom’s level:** Application

**Question 5**

Which clinical finding is the **most reliable early indicator** of opioid-induced ventilatory impairment in this patient?

A. Increasing sedation associated with a declining respiratory rate
B. Mild nausea after PCA initiation
C. Tachycardia with preserved alertness
D. Mild postoperative hypertension

**Correct answer:** A
**Bloom’s level:** Analysis

**Clinical Scenario 2**

**Multiple Rib Fractures with Pneumothorax**

A **56-year-old man** presents after a road traffic collision with **multiple rib fractures and pneumothorax**. He has severe chest pain and avoids deep breathing and coughing because of pain.

**Question 6**

What is the **primary clinical objective** of providing effective analgesia in this patient?

A. To reduce sympathetic stimulation and anxiety
B. To improve ventilation and reduce the risk of pulmonary complications
C. To achieve complete absence of pain at rest
D. To lower blood pressure caused by trauma

**Correct answer:** B
**Bloom’s level:** Understanding

**Question 7**

Which analgesic technique is most likely to provide **superior pain control and improved respiratory mechanics** in this patient with multiple rib fractures?

A. Thoracic epidural analgesia
B. Oral acetaminophen alone
C. Intermittent intramuscular opioids
D. Continuous sedative infusion

**Correct answer:** A
**Bloom’s level:** Application

**Question 8**

On the following day, the patient develops shallow breathing, poor cough effort, and reduced air entry at the lung bases. Which complication is most likely related to **inadequately controlled rib fracture pain**?

A. Pulmonary embolism due to immobility
B. Atelectasis with secondary chest infection
C. Opioid-induced chest wall rigidity
D. Tension pneumothorax caused by coughing

**Correct answer:** B
**Bloom’s level:** Analysis

**Question 9**

A gabapentinoid is considered as part of a multimodal regimen. Which mechanism best explains its analgesic contribution?

A. Cyclooxygenase inhibition and reduced prostaglandin synthesis
B. Modulation of voltage-gated calcium channels, reducing excitatory neurotransmitter release
C. Direct activation of mu-opioid receptors
D. Sodium channel blockade at the peripheral nerve membrane

**Correct answer:** B
**Bloom’s level:** Understanding

**Question 10**

Which analgesic delivery strategy best allows **patient-controlled opioid administration within predefined safety limits**?

A. Nurse-administered PRN opioid dosing
B. Intravenous patient-controlled analgesia (IV PCA)
C. Continuous background opioid infusion without bolus restriction
D. Fixed-schedule oral opioid dosing every 12 hours

**Correct answer:** B
**Bloom’s level:** Application

**Clinical Scenario 3**

**Severe Postoperative Pain After Total Knee Replacement**

A **73-year-old patient with coronary artery disease and previous coronary artery bypass grafting (CABG)** undergoes total knee replacement. Four hours postoperatively, the patient reports **severe knee pain (NRS 9/10)**.

**Question 11**

Which regional analgesic technique is most appropriate as part of postoperative pain management after **total knee arthroplasty**?

A. Femoral nerve block or adductor canal block
B. Stellate ganglion block
C. Intercostal nerve block
D. Lumbar sympathetic block

**Correct answer:** A
**Bloom’s level:** Application

**Question 12**

Which statement best explains why **multimodal analgesia** is recommended after major orthopedic surgery?

A. It replaces the need for postoperative monitoring
B. It reduces opioid requirements by targeting pain through different mechanisms
C. It eliminates cardiovascular risk associated with analgesics
D. It provides effective pain relief primarily by increasing sedation

**Correct answer:** B
**Bloom’s level:** Understanding

**Question 13**

The surgical team considers NSAIDs for opioid-sparing analgesia. In this patient with established **cardiovascular disease**, which consideration is most important?

A. NSAIDs may increase the risk of cardiovascular thrombotic events
B. NSAIDs commonly cause profound respiratory depression
C. NSAIDs are contraindicated only in patients with asthma
D. NSAIDs are ineffective in postoperative orthopedic pain

**Correct answer:** A
**Bloom’s level:** Application

**Question 14**

The patient is started on **IV PCA opioids**. Which monitoring parameter is most important for early detection of clinically significant opioid-related harm?

A. Body temperature and capillary refill
B. Respiratory rate and level of sedation
C. Urine output and fluid balance
D. Blood glucose and blood pressure

**Correct answer:** B
**Bloom’s level:** Application

**Question 15**

When selecting the most appropriate postoperative analgesic strategy for this patient, which factor should carry the **greatest overall weight**?

A. Medication acquisition cost alone
B. Surgical procedure, pain severity, and patient comorbidities
C. Standard ward prescribing habits
D. Patient age in isolation

**Correct answer:** B
**Bloom’s level:** Analysis

**Instrument Blueprint**

| **Domain** | **Items** |
| --- | --- |
| Pain assessment | 2 |
| Multimodal analgesia | 4 |
| Opioid safety & monitoring | 3 |
| Regional anesthesia | 2 |
| Adjuvant analgesics | 1 |
| Respiratory consequences | 2 |
| Patient-specific decisions | 1 |

**Bloom’s Taxonomy**

| **Level** | **Items** |
| --- | --- |
| Understanding | 4 |
| Application | 8 |
| Analysis | 3 |

**Reliability & Validity**

- Cronbach’s alpha = 0.81
- CVI (S-CVI/Ave) = 0.97
- Pilot tested (n = 12 students)

**SUPPLEMENTARY FILE 2**

**Acute Pain Management Learner Confidence Scale (APMLCS)**

**Instrument Overview**

The APMLCS measures self-reported confidence in acute pain management competencies among final-year medical students following simulation-based education.

**Structure**

- 8 items
- 5-point Likert scale
- Score range: 8–40

**Response Scale**

1 = Strongly disagree
2 = Disagree
3 = Neutral
4 = Agree
5 = Strongly agree

**Items**

1. Pain assessment using validated tools
2. Multimodal analgesia selection
3. Analgesic drug selection based on patient condition
4. Recognition of opioid respiratory risk
5. Monitoring opioid-related complications
6. Identifying need for regional analgesia
7. Considering comorbidities in analgesic choice
8. Developing multimodal pain plans

**Scoring Interpretation**

| **Score** | **Level** |
| --- | --- |
| 8–16 | Low |
| 17–24 | Moderate |
| 25–32 | High |
| 33–40 | Very high |

**Psychometrics**

- Cronbach’s alpha = 0.84
- CVI (S-CVI/Ave) = 0.96
- Pilot tested (n = 12)

**SUPPLEMENTARY FILE 3**

**Simulation Learning Satisfaction Scale (SLSS)**

**Instrument Overview**

The SLSS evaluates student satisfaction with high-fidelity simulation training in acute pain management.

**Structure**

- 6 items
- 5-point Likert scale
- Score range: 6–30

**Response Scale**

1 = Strongly disagree
2 = Disagree
3 = Neutral
4 = Agree
5 = Strongly agree

**Items**

1. Realism of simulation scenarios
2. Improved understanding of pain management
3. Integration of theory and practice
4. Safe clinical decision-making environment
5. Quality of feedback and debriefing
6. Overall educational value

**Scoring Interpretation**

| **Score** | **Level** |
| --- | --- |
| 6–12 | Low |
| 13–18 | Moderate |
| 19–24 | High |
| 25–30 | Very high |

**Psychometrics**

- Cronbach’s alpha = 0.86
- CVI (S-CVI/Ave) = 0.95
- Pilot tested (n = 12)
